# Cross-sectional associations between physical activity and sedentary time with cardiovascular health in children from the ALSPAC study using compositional data analysis

**DOI:** 10.1038/s41598-025-95407-x

**Published:** 2025-04-07

**Authors:** K. M. Sansum, B. Bond, R. M. Pulsford, A. McManus, A. O. Agbaje, A. M. Skinner, A. R. Barker

**Affiliations:** 1 Children’s Health and Exercise Research Centre, Public Health and Sport Sciences, University of Exeter Medical School, University of Exeter, Exeter, UK; 2 Physical Activity and Health Across the Lifespan, Public Health and Sport Sciences, University of Exeter Medical School, University of Exeter, Exeter, UK; 3 Centre for Heart, Lung and Vascular Health, School of Health and Exercise Sciences, University of British Columbia, Kelowna, British Columbia Canada; 4 Institute of Public Health and Clinical Nutrition, School of Medicine, Faculty of Health Sciences, University of Eastern Finland, Kuopio, Finland

**Keywords:** Cardiovascular biology, Risk factors

## Abstract

This study adopted a compositional framework to cross-sectionally examine the associations between physical activity (PA) and sedentary time (ST) with vascular structure and function and clustered cardiovascular disease (CVD) risk factors in 4277 children (2,226 girls), aged 10.6±0.2 years. Cardiovascular outcomes included flow mediated dilation, distensibility coefficient, pulse wave velocity and a clustered CVD risk factor score. Time spent in light PA (LPA) and moderate to vigorous PA (MVPA) and ST were determined using accelerometers. Multiple linear regression analyses were adjusted for key covariates with LPA, MVPA and ST entered as compositional exposure variables. Neither LPA, MVPA or ST were significantly associated with the vascular outcomes. The proportion of time spent in MVPA and ST were inversely (unstandardised b=−0.126; *P*=0.001) and positively (b=0.136; *P*=0.016) associated with clustered CVD risk in the whole group analysis, respectively. MVPA was negatively associated with clustered CVD risk in boys (b=−0.144; *P*=0.011) and girls (b=−0.110; *P*=0.032). Only girls had a positive association between ST and clustered CVD risk (b=0.199; *P=*0.005). Although no associations were observed for PA and ST with vascular outcomes, these data provide further support for interventions that promote MVPA and minimise ST for reducing risk factors for CVD in children.

## Introduction

Previous research has shown a relationship between traditional cardiovascular disease (CVD) risk factors (e.g. adiposity and hypertension) and arterial outcomes in children and adolescents^1^, underscoring the importance of the primary prevention or modification of CVD risk factors in this population^2^. While extensive literature has been published regarding the relationship between physical activity (PA) and sedentary time (ST) with traditional CVD risk factors in children and adolescents^3,4^, few studies have examined associations with measures of arterial structure and function.

A review by Baumgartner et al.^5^ identified several studies reporting favourable associations between PA and vascular outcomes, although significant associations were more common in studies employing self-report measures of PA. However, self-report measures of PA are subject to concerns with measurement properties and recall and reporting bias^6^, when compared to studies using device-based measures, which may mask or alter associations with health outcomes. Regarding ST, the majority of observational studies report null associations between ST and vascular outcomes^7–12^. An exception is the study by Fujiwara et al.^13^ which observed associations between a two year change in arterial stiffness and behaviours associated with ST (i.e. watching television) in adolescents, although there was a lack of control for important confounders, including PA, cardiorespiratory fitness (CRF) and body composition. Therefore, while there is emerging evidence concerning the association between PA and ST with vascular outcomes in children and adolescents, the extant data is considered to be of low quality, with studies limited by small sample sizes from predominantly European countries, failing to control for important confounders and not employing device-determined measures of PA^5,14^. Furthermore, the small sample sizes have resulted in few studies (e.g. Fujiwara et al.^13^) examining the potential sex differences in the associations.

A further methodological concern is that previous literature has not considered the unique properties of time-based measures of PA and ST when examining association with vascular outcomes in children and adolescents. When measured in minutes per day, PA and ST data are compositional in nature so the parts sum to total a finite amount of time (e.g. 16 waking hours if we assume 8 hours of sleep)^15^, and are comprised of mutually exclusive parts^16^. For example, a change in the time spent in one intensity of PA (e.g. moderate to vigorous intensity PA [MVPA]) will alter the time spent in one or more of the other intensities of PA (i.e. light intensity PA [LPA] and/or ST)^17^. As time spent in different intensities of PA and ST are co-dependent, they should be considered as a whole when examining associations with health outcomes^18^. The standard regression model approach used in the existing literature may include one of the other activity behaviours in the model as a covariate (e.g. adjusting for ST when examining associations between MVPA and the outcome variable) but cannot also include the remaining activity behaviour (LPA) or it would violate the assumption of collinearity and result in biased estimates. A statistical approach which considers the unique properties of time-based activity data is compositional data analysis (CoDA)^16^. This approach allows all activity behaviours (ST, LPA and MVPA) to be added into the same model without violating the collinearity assumption and has been successfully applied in paediatric data to examine the associations between PA and ST with single and clustered risk factors for CVD^19–21^. However, CoDA has yet to be applied to examine associations between PA and ST with vascular outcomes in children.

The primary aim of this study was to use CoDA to examine the associations between device-derived LPA, MVPA and ST with endothelial function, arterial elasticity, arterial stiffness in a population sample of children, and to investigate whether these associations differ with the data stratified by sex. Furthermore, as vascular structure and function are altered in the presence of traditional CVD risk factors in children and adolescents, a secondary aim was to examine associations between LPA, MVPA and ST with clustered CVD risk.

## Methods

### Participants

The Avon Longitudinal Study of Parents and Children (ALSPAC) is a large prospective cohort study from Bristol, UK^22,23^. Ethical approval for the study was obtained from the ALSPAC Ethics and Law Committee and the Local Research Ethics Committees. Consent for biological samples has been collected in accordance with the Human Tissue Act (2004). Informed consent for the use of data collected via questionnaires and clinics was obtained from participants following the recommendations of the ALSPAC Ethics and Law Committee at the time. Full details of the cohort and study design have been published previously (www.alspac.bris.ac.uk)^24^. Please note that the study website contains details of all the data that is available through a fully searchable data dictionary and variable search tool (http://www.bristol.ac.uk/alspac/researchers/our-data/).

Pregnant women resident in Avon, UK with expected dates of delivery between 1st April 1991 and 31st December 1992 were invited to take part in the study. The initial number of pregnancies enrolled was 14,541 and 13,988 children were alive at 1 year of age. When the oldest children were approximately 7 years of age, an attempt was made to bolster the initial sample with eligible cases who had failed to join the study originally. The total sample size for analyses using any data collected after the age of seven was 15,447 pregnancies. Of these, 14,901 children were alive at 1 year of age. Participants were included in the current study if they had complete vascular data at age 10 years and valid accelerometer data at age 11 years, resulting in a sample of 4,277 children.

### Physical activity and sedentary time data

ActiGraph accelerometers (AM7164, 2.2, ActiGraph LLC, Fort Walton Beach, FL, USA) were used to determine PA and ST. Participants were asked to wear the accelerometers during their waking hours on their right hip for seven consecutive days at age 11 years old. The device was removed when bathing or when participating in water-based activities. Movement acceleration was aggregated over 60 s sampling periods, and data were expressed in counts⋅min^−1^.

Kinesoft (V.3.3.75; Kinesoft, Saskatchewan, Canada) was used to process the accelerometer data^25^. A valid day was defined as a minimum of 500 min of data following the exclusion of ≥60 min of zero counts, and allowing up to two min of interruptions^25^. However, previous research has recommended a minimum wear time of 10 h∙day^−1^ for children to achieve a good estimate of habitual PA^26,27^. To align the inclusion criteria closer to this, the sample in the present study was further restricted to participants who had an average valid wear time of 600 min⋅day^−1^. Participants also needed at least three days of valid data^25^ but this was not constrained to include a weekend day.

Although there are specific cut-points derived for the ALSPAC dataset^28^, the MVPA cut-point is considerably higher than other thresholds derived for children of the same age such as those derived by Evenson et al.^29^ (>3,600 counts⋅min^-1^ vs. >2296 counts⋅min^−1^, respectively), and may underestimate MVPA. Furthermore, the ALSPAC cut-point for ST (<200 counts∙min^−1^) was not defined in the original validation study^28^.This threshold for ST is higher than the <100 counts∙min^−1^ used in previous literature^29,30^ and may artificially inflate estimates of ST. Therefore, the Evenson et al.^29^ thresholds were used as recommended previously for children and adolescents^31^.

### Vascular outcomes

#### Endothelial function

Endothelial function was assessed via flow mediated dilation (FMD) of the brachial artery at age 10 years. Details of the full protocol have been published previously^32^. Briefly, high resolution (7 MHz) ultrasound (ALOKA 5500, Keymed, UK) was used to image a straight, non-branching section of the right brachial artery, 5–10 cm above the antecubital fossa. Baseline brachial artery diameter was recorded for 1 min before a pneumatic cuff was inflated around the forearm (immediately distal to the medial epicondyle) to 200 mmHg for 5 min. The cuff was rapidly deflated with an automatic air regulator (Logan Research, UK) and recording continued for a further 3 min to capture peak dilation of the artery. In adults, when performed in this way, FMD is considered to be nitric oxide mediated^33^. End-diastole electrocardiogram triggered images were captured at 3 s intervals throughout the protocol. Brachial artery diameter was measured using edge-detection software (Brachial Tools, Medical Imaging Applications, USA). The FMD (%) response was calculated using the equation:

$$\text{FMD} (\%) = \frac{\text{peak diameter} - \text{baseline diameter}}{\text{baseline diameter}} \times 100$$

where, diameter measurements are expressed in mm, as quantified using the edge detection software.

Between day coefficients of variation (%) for baseline diameter and FMD (%) were 4.9% and 10.9%, respectively. Additionally, reliability of the image analysis had an inter- and intra- observer variability of 1.6% and 1.2% for baseline diameter, and 5.6% and 5.3% for FMD (%)^32^.

#### Arterial elasticity

Brachial artery distensibility was assessed using high resolution ultrasound and arterial blood pressure. B-mode ultrasound images (ALOKA 5500, Keymed, UK) of the same segment of the brachial artery used for FMD analysis were recorded for 20 s and simultaneously blood pressure was recorded in the contralateral arm using an oscillometric blood pressure device (Omron MI-5). Arterial distension was calculated as the luminal diameter excursion from diastole to systole. The distensibility coefficient (DC, indication of vascular wall elasticity) was derived from the pulse pressure and distension of the artery and expressed as mean percentage change in cross-sectional area per unit change in blood pressure and had a coefficient of variation of 18%^32^.

#### Arterial stiffness

Pulse wave velocity (PWV) provided a measure of arterial stiffness at age 10 years. A high-fidelity micromanometer (SPC-301, Millar Instruments, Houston, TX, USA) transcutaneously recorded pressure-pulse waveforms from the radial and carotid pulse using synchronous ECG, to provide an R-timing reference. Data were processed using integral software (SphygmoCor version 7.1, Scanmed, UK) to calculate the mean time difference between the R-wave and pressure wave on a beat-to-beat basis over 10 s. The PWV was determined by arterial path length between the carotid and radial arteries (m)/ mean time difference between R wave and pressure wave (s). The coefficient of variation for PWV was 8.7%^32^.

### Anthropometry

At age 9 and 10 years, stature was measured to the nearest 0.1 cm (Harpenden Stadiometer) and body mass to the nearest 0.05 kg (Tanita Body Fat Analyser; Model TBF305). Body mass index (BMI, kg⋅m^−2^) was calculated, and the age and sex-specific standard deviation score for BMI was calculated from the 1990 British Growth Reference charts^34^.

### Body composition

Fat and lean mass were assessed at 9 years using A Lunar Prodigy dual-energy X-ray absorptiometry (DEXA) scanner (GE Medical Systems, Chalfont St Giles, UK). Body composition data assessed via DEXA are considered valid and reliable with coefficients of variation between 0.01 and 4.37%^35^. Total body fat and lean mass indices were calculated using fat or lean mass (kg)⋅height^−2^ (m), with the stature variable also taken from the clinic at 9 years. These methods of normalising were chosen to account for variation in body size between participants that could influence the measures of body composition^36–38^.

### Pubertal status

Superimposition by Translation and Rotation mixed-effects growth curve analysis^39^ was used to determine the age at peak height velocity^40^. The age in years to peak height velocity (aPHV) at both the 9 and 10 years clinics was taken as an objective measure of pubertal (somatic) status.

### Blood pressure

Blood pressure was recorded in the right arm at age 9 years using a Dinamap 9301 Vital Signs Monitor (Morton Medical, London, United Kingdom) whilst participants were in the seated position. The mean of two recordings was used in analysis. Mean arterial pressure (MAP) was derived from diastolic blood pressure + 1/3(systolic—diastolic blood pressure).

### Cardiorespiratory fitness

The physical work capacity at a heart rate of 170 beats∙min^-1^ (PWC_170_) was used as an indicator of CRF measured in W at age 9 years^41^. In brief, participants completed an exercise test on a cycle ergometer (Lode Rechor P), consisting of 3 stages of increasing workload (20, 40 and 60 W). Heart rate was recorded every 5-s during the test via a chest strap (Polar S180). The mean heart rate obtained during the final minute of each stage was used via linear regression to predict the workload required to elicit a heart rate of 170 beats∙min^−1^. The test was considered successful if the child achieved a heart rate of >80 beats∙min^−1^ during the first exercise stage, indicating an appropriate response to the workload, and >150 beats∙min^−1^ by stage three. The criteria of attaining >150 beats∙min^−1^ is approximately equivalent to a workload of >70% of maximum heart rate and was used to ensure that all children exercised at a power output that elicited a heart rate close to the predicted value of 170 beats∙min^−1^. The PWC_170_ test has moderate criterion validity with cycling determined absolute peak oxygen uptake (r = 0.49–0.54; *P* < 0.01) in 11–16 year olds^42^.

To control for the influence of body size and composition, CRF data (W) were ratio and allometrically scaled (using log-linear regression models) for body mass and lean mass^43^. Sex and body mass or lean body mass were entered as the independent variables and PWC_170_ (W) as the dependent variable in the regression models. The validity of the scaling methods to remove the influence of body size was confirmed by correlating the scaled CRF against the relevant body size variables at both a group level and with the data stratified by sex using Pearson’s correlation coefficient.

### Blood outcomes

During the clinic aged 9 years, non-fasted blood samples were collected using standardised procedures. Blood samples were spun immediately, and plasma was stored at −80 ℃. Plasma lipids (total cholesterol, triglycerides (TAG), high density lipoprotein (HDL)) were analysed using enzymatic reagents^44^ and insulin concentration was determined with an enzyme-linked immunosorbent assay (Mercodia, Uppsala, Sweden). The coefficient of variation for all assays was <5%.

### Family history of disease

Participants were asked to report their family history of hypertension, diabetes, high cholesterol and vascular disease.

### Clustered cardiovascular disease risk

A clustered risk factor score for CVD was comprised from variables assessed at age 9 years: fat mass index, MAP, TAG, total cholesterol: HDL ratio and insulin concentration. Variables were log-transformed for analysis due to account for the skewed distribution. The sex-specific z-scores for each variable were calculated, and the sum of these z-scores was divided by 5 (the number of variables) in line with previous studies^45^.

### Missing data

Missing data (listwise deletion) were present for most variables (Supplementary Table 1). Little’s missing cases at random test (performed in SPSS, v.26) showed that data were missing at random (*P*<0.001) and therefore, imputation of missing data was performed using the multiple imputation by chained equations (mice) package (v 3.12.0) in RStudio (http://cran.r-project.org). It was estimated that 20 cycles of imputations with 10 iterations via regression models would be sufficient based on the percentage of missing values for variables^46,47^. Data from the imputation cycles was pooled for analysis. For the variable with the largest percentage of missing data (mother’s social class, 54.4% missing), the estimate using Rubin’s formula, was that the pooled values were 97% efficient after 20 imputations.

After imputation, data were examined to check the imputed values maintained a similar distribution as the observed values. Supplementary Table 2 presents the variables that were imputed along with the minimum and maximum values and Supplementary Table 3 presents summary statistics (Mean, SD, percentages) for the observed vs. imputed values. The imputed form of the analysis is presented in the main text of the paper but a sensitivity analysis using the observed data is provided as a supplement.

### Statistical analyses

Data are reported as mean ± standard deviation (SD) and b coefficients with 95% confidence intervals (CI), unless otherwise stated. The null hypothesis was rejected at an alpha level of 0.05. Descriptive statistics were analysed using SPSS statistics (version 26, IBM, Armonk, NY) and RStudio. Data were checked for normality using the Shapiro-Wilk test and via visual inspection of histograms. Mann-Whitney-U tests were used to assess sex differences in baseline characteristics for continuous variables due to the assumption of normality being violated (apart from aPHV at 9 years clinic where an independent t-test was used) and chi-squared tests for categorical variables.

The principles and theory behind using the CoDA approach for analysing time spent in activity behaviour categories have been described in detail elsewhere^15,17,18^. Multiple linear regression analyses were run in RStudio with either FMD, PWV, DC or clustered CVD risk as the outcome variable and the relevant pair of isometric log ratio (*ilr*) co- ordinates as the predictor variable. The regression models were created *a priori* based on reviews of the existing literature^5,14^ and established confounding variables^32,48–51^. Analyses were adjusted for age at 10 years clinic (or 9 years when clustered CVD risk was the outcome), sex (in whole group models only), age in years from peak height velocity at 10 years clinic (or 9 years when clustered CVD risk was the outcome), mother’s social class (I to V), baseline vessel diameter (mm; only when FMD was the outcome), the time between vascular and accelerometer measurements (years) or time between clustered CVD risk and accelerometer measurements (years), CRF scaled to lean body mass (W⋅kg^0.59^), the lean mass index (kg⋅m^−2^), clustered CVD risk (apart from when clustered CVD risk was the outcome), and family history of disease.

The multiple linear regression models were also run with the inclusion of an interaction term between sex and the activity behaviour variable to examine if there was a significant difference in the associations between the boys and the girls. No models had a statistically significant interaction term (data not presented). Regressions are presented for the whole group and with the data stratified by sex due to observed behavioural and biological sex differences in the variables (Table 1). All regression models were confirmed to meet assumptions for linearity, normality and homoscedasticity of residuals and no multicollinearity. The results section presents the fully adjusted regression models, but the unadjusted models are presented in Supplementary Table 5.

**Table 1:** Demographics and confounding variables for the whole group and when stratified by sex.

	Group	*n*	Boys	*n*	Girls	*n*	*P*-value
Age at 9-year clinic (y)	9.8 ± 0.3	4089	9.8 ± 0.3	1959	9.8 ± 0.3	2130	0.69
Age at 10-year clinic (y)	10.6 ± 0.2	4277	10.6 ± 0.2	2051	10.6 ± 0.2	2226	0.84
aPHV at 9 y (y)	−2.8 ± 1.28	3370	−3.8 ± 0.9	1569	−2.0 ± 0.9	1801	**< 0.001**
aPHV at 10 y (y)	−2.0 ± 1.3	3449	−3.0 ± 0.9	1609	−1.2 ± 0.9	1840	**< 0.001**
Body mass at 9 y (kg)	34.3 ± 7.1	4080	34.0 ± 6.7	1954	34.5 ± 7.4	2126	0.09
Body mass at 10 y (kg)	37.6 ± 8.2	4261	37.2 ± 7.8	2044	38.0 ± 8.5	2217	**0.004**
Stature at 9 y (cm)	139.3 ± 6.2	4043	139.6 ± 6.0	1944	139.0 ± 6.4	2099	**< 0.001**
Stature at 10 y (cm)	143.8 ± 6.6	4247	143.7 ± 6.3	2040	143.8 ± 6.8	2207	0.68
BMI at 10 y (kg⋅m^−2^)	18.1 ± 3.0	4240	17.9 ± 2.7	2038	18.2 ± 3.1	2202	**< 0.001**
BMI SD at 10 y	0.27 ±1.14	4240	0.34 ± 1.13	2038	0.20 ± 1.14	2202	**< 0.001**
Mother’s social class							0.06
I—Professional (%)	5.5	107	6.5	61	4.5	46	
II—Managerial & technical (%)	36.1	703	35.4	331	36.6	372	
III—Skilled, non-manual (%)	38.9	758	38.1	356	39.6	402	
III Skilled, manual (%)	1.5	30	0.9	8	2.2	22	
IV—Partly unskilled (%)	14.7	287	15.4	144	14.1	143	
V—Unskilled (%)	3.3	65	3.6	34	3.1	31	
Body composition measures at 9 year clinic							
Total body fat mass (kg)	8.32 ± 4.85	3903	7.16 ± 4.56	1870	9.38 ± 4.86	2033	**< 0.001**
Fat mass index (kg⋅m^−2^)	4.22 ± 2.30	3863	3.61 ± 2.15	1859	4.79 ± 2.28	2004	**< 0.001**
Total body lean mass (kg)	24.40 ± 3.17	3903	25.39 ± 2.88	1870	23.49 ± 3.15	2033	**< 0.001**
Lean mass index (kg⋅m^−2^)	12.53 ± 0.97	3863	12.97 ± 0.84	1859	12.12 ± 0.90	2004	**< 0.001**
Fitness measure at 9 year clinic							
PWC_170_ (W)	64 ± 9	2084	67 ± 8	866	62 ± 9	1218	**< 0.001**
PWC_170_⋅total body mass (W⋅kg^−1^)	1.9 ± 0.4	2081	2.1 ± 0.4	865	1.8 ± 0.3	1216	**< 0.001**
PWC_170_⋅total body mass (W⋅kg^0.24^)	27.8 ± 3.8	2081	29.3 ± 3.5	865	26.8 ± 3.7	1216	**< 0.001**
PWC_170_⋅total body lean mass (W⋅kg^0.59^)	10.0 ± 1.3	1991	10.3 ± 1.2	824	9.8 ± 1.3	1167	**< 0.001**
Metabolic profile at 9 year clinic							
Cholesterol (mmol⋅L^−1^)	4.27 ± 0.63	2778	4.21 ± 0.63	1355	4.34 ± 0.63	1423	**< 0.001**
HDL (mmol⋅L^−1^)	1.41 ± 0.31	2778	1.44 ± 0.31	1355	1.37 ± 0.30	1423	**< 0.001**
Total cholesterol:HDL ratio	3.17 ± 0.79	2778	3.03 ± 0.74	1355	3.29 ± 0.81	1423	**< 0.001**
TAG (mmol⋅L^−1^)	1.12 ± 0.52	2778	1.10 ± 0.53	1355	1.13 ± 0.52	1423	**0.008**
*Insulin (mU⋅L^−1^)	8.03 (10.39)	2760	8.01 (11.11)	1348	8.03 (9.87)	1412	0.52
Cardiometabolic risk score	−0.01 ± 0.61	2546	0.00 ± 0.60	1250	0.02 ± 0.61	1296	0.24
Family history of hypertension, diabetes, high cholesterol and vascular disease							0.48
Yes (%)	30.2	830	29.5	353	30.8	477	
No (%)	69.8	1914	70.5	842	69.2	1072	
Time gap between measures							
Time between vascular and accelerometer visits (y)	1.1 ± 0.3	4277	1.1 ± 0.3	2051	1.1 ± 0.3	2226	0.53
Time between CVD score and vascular visits (y)	0.8 ± 0.3	4089	0.8 ± 0.3	1959	0.8 ± 0.3	2130	0.61
Time between CVD score and accelerometer visits (y)	1.9 ± 0.3	4089	1.9 ± 0.3	1959	1.9 ± 0.3	2130	0.38

Data presented as mean ± SD. * indicates median (interquartile range). Significant values are in bold.

*aPHV* age in years from peak height velocity, *BMI* body mass index, *PWC*_*170*_ peak work capacity at 170 beats per minute, *HDL* high density lipoprotein, *TAG* triglyceride.

## Results

Demographic and confounding variable data are presented in Table 1. There were sex differences in all variables apart from the age of participants at the clinics at age 9 and 10 years, body mass age 9 years, stature age 10 years, mother’s social class, insulin concentration, family history of hypertension, diabetes, high cholesterol and vascular disease, time between visits to the vascular and accelerometer clinics, time between clustered CVD risk and vascular clinics, and the time between the clustered CVD risk and accelerometer clinics.

Vascular and accelerometer variables are presented in Table 2. There were sex differences in all variables apart from SBP at age 9 years and time spent in LPA. For the vascular variables, girls were characterised by a higher blood pressure (systolic, systolic and MAP) and FMD (%) and a lower DC and PWV compared to boys. Girls also had a greater ST and a lower MVPA compared to boys.

**Table 2:** Vascular and accelerometer variables for the whole group and when stratified by sex.

	Group	*n*	Boys	*n*	Girls	*n*	*P*-value
Vascular variables							
Systolic blood pressure age 9 y (mmHg)	102 ± 9	4045	102 ± 9	1941	102 ± 9	2104	0.30
Diastolic blood pressure age 9 y (mmHg)	57 ± 6	4046	57 ± 6	1941	58 ± 6	2105	**0.014**
MAP age 9 y	72 ± 6	4045	72 ± 6	1941	73 ± 6	2104	**0.034**
Systolic blood pressure age 10 y (mmHg)	104 ± 9	4277	103 ± 9	2051	104 ± 9	2226	**0.019**
Diastolic blood pressure age 10 y (mmHg)	60 ± 8	4277	60 ± 7	2051	61 ± 8	2226	**< 0.001**
Baseline vessel diameter (mm) age 10 y	2.66 ± 0.30	4277	2.75 ± 0.29	2051	2.58 ± 0.29	2226	**< 0.001**
FMD absolute (mm) age 10 y	0.21 ± 0.08	4277	0.21 ± 0.08	2051	0.22 ± 0.08	2226	**0.039**
FMD (%) age 10 y	8.13 ± 3.39	4277	7.75 ± 3.28	2051	8.48 ± 3.45	2226	**< 0.001**
DC (% per mmHg) age 10 y	0.12 ± 0.06	4277	0.13 ± 0.06	2051	0.12 ± 0.06	2226	**< 0.001**
PWV (m⋅s^−1^) age 10 y	7.56 ± 1.23	4277	7.64 ± 1.24	2051	7.48 ± 1.20	2226	**< 0.001**
Accelerometer variables							
Accelerometer wear time (min⋅day^−1^)	778.5 ± 59.6	4277	783.3 ± 60.6	2051	774.2 ± 58.4	2226	**< 0.001**
ST (min⋅day^−1^)	354.5 ± 72.6	4277	347.6 ± 73.6	2051	360.8 ± 71.2	2226	**< 0.001**
LPA (min⋅day^−1^)	366.5 ± 59.5	4277	367.4 ± 59.6	2051	365.6 ± 59.4	2226	0.24
MVPA (min⋅day^−1^)	57.6 ± 29.6	4277	68.2 ± 32.3	2051	47.7 ± 22.8	2226	**< 0.001**

Data presented as mean ± SD. Significant values are in bold.

*MAP* mean arterial pressure, *FMD* flow mediated dilation, *PWV* pulse wave velocity, *DC* distensibility coefficient.

Table 3 presents the compositional geometric means of the PA and ST data for the group, and with the data stratified by sex. At a group level, the greatest portion of time was spent in LPA (47.6%), followed by ST (45.7%). Time spent performing MVPA only accounted for a small portion of time (6.7%). Boys and girls spent a similar proportion of time in LPA, but girls spent a larger proportion of the time sedentary compared to boys and performed proportionally less MVPA. The robust variance matrices for the compositional models are provided in Supplementary Table 4.

**Table 3:** Geometric means of activity behaviours for whole group, boys and girls.

	Group (*n* = 4277)		Boys (*n* = 2051)		Girls (*n* = 2226)	
	min⋅day^−1^	%	min⋅day^−1^	%	min⋅day^−1^	%
ST	438.4	45.7	426.9	44.5	448.2	46.7
LPA	457.2	47.6	455.9	47.5	457.3	47.6
MVPA	64.5	6.7	77.2	8.0	54.4	5.7

Data are presented as the geometric mean and the percentage of the participant’s waking time.

*ST* sedentary time, *LPA* light physical activity, *MVPA* moderate to vigorous physical activity.

The raw unadjusted models for the association between the activity behaviours and the vascular and clustered CVD risk outcomes are presented in Supplementary Table 5. The fully adjusted regression models identified no significant associations between the activity behaviours (LPA, MVPA and ST) and vascular outcomes (FMD (%), DC and PWV) in the group models, or when stratified by sex (Table 4). However, MVPA was inversely associated with the clustered CVD risk in the whole group model and when stratified for girls and boys. In addition, ST was positively associated with clustered CVD risk in the group model and girls, but was no association was observed for boys. Finally, LPA was not associated with the clustered CVD risk in the group model or when the data was stratified by sex.

**Table 4:** Associations between activity behaviours with endothelial function, arterial stiffness, arterial elasticity and clustered cardiovascular risk using compositional data analysis for accelerometer data.

	Group		Boys		Girls	
	β_i*lr1*_ value (95% CI)	*P*-value	β _*lr1*_ value (95% CI)	*P*-value	β _*lr1*_ value (95% CI)	*P*-value
Endothelial function						
ST: LPA & MVPA	0.046 (−0.327 to 0.418)	0.81	−0.230 (−0.741 to 0.282)	0.37	0.305 (−0.234 to 0.844)	0.26
LPA: MVPA & ST	−0.026 (−0.502 to 0.449)	0.91	0.221 (−0.442 to 0.885)	0.50	−0.248 (−0.929 to 0.433)	0.47
MVPA: ST & LPA	−0.019 (−0.290 to 0.252)	0.89	0.008 (−0.373 to 0.389)	0.97	−0.057 (−0.441 to 0.327)	0.77
Arterial stiffness						
ST: LPA & MVPA	0.054 (−0.086 to 0.194)	0.44	0.106 (−0.095 to 0.308)	0.29	0.009 (−0.185 to 0.202)	0.93
LPA: MVPA & ST	−0.091 (−0.269 to 0.087)	0.31	−0.230 (−0.492 to 0.031)	0.08	0.036 (−0.209 to 0.281)	0.77
MVPA: ST & LPA	0.036 (−0.065 to 0.138)	0.48	0.124 (−0.025 to 0.273)	0.10	−0.044 (−0.183 to 0.095)	0.52
Arterial elasticity						
ST: LPA & MVPA	−0.004 (−0.011 to 0.003)	0.29	−0.004 (−0.014 to 0.006)	0.39	−0.003 (−0.013 to 0.007)	0.53
LPA: MVPA & ST	0.005 (−0.004 to 0.014)	0.26	0.004 (−0.009 to 0.017)	0.50	0.006 (−0.006 to 0.018)	0.35
MVPA: ST & LPA	−0.001 (−0.006 to 0.004)	0.58	−8.61E−05 (−0.008 to 0.007)	0.98	−0.003 (−0.010 to 0.004)	0.44
Clustered CVD risk score						
ST: LPA & MVPA	**0.136 (0.026 to 0.246)**	**0.016**	0.072 (−0.074 to 0.219)	0.32	**0.199 (0.060 to 0.339)**	**0.005**
LPA: MVPA & ST	−0.010 (−0.135 to 0.115)	0.88	0.072 (−0.122 to 0.266)	0.46	−0.089 (−0.250 to 0.071)	0.27
MVPA: ST & LPA	**−0.126 (−0.202 to −0.050)**	**0.001**	**−0.144 (−0.255 to −0.034)**	**0.011**	**−0.110 (−0.211 to −0.009)**	**0.032**

Models were adjusted for age at 10 years clinic (y; or 9 years when CVD was outcome), sex (in whole group models only), age in years from peak height velocity at 10 year clinic (years; or 9 years when CVD was outcome), mother’s social class (I to V), baseline vessel diameter (mm; only when FMD was the outcome), the time between vascular and accelerometer measurements (years) or time between CVD score and accelerometer measurements (years), cardiorespiratory fitness scaled to lean body mass (W⋅kg^0.59^), the lean mass index (kg⋅m^-2^), Clustered CVD risk score (apart from when CVD risk score was the outcome), and family history of hypertension, diabetes, high cholesterol, and vascular disease. Significant values are in bold.

*ST* sedentary time, *LPA* light physical activity, *MVPA* moderate to vigorous physical activity, *CVD* cardiovascular disease risk.

A sensitivity analysis was performed using the observed data (Supplementary Table 6). This provided the same findings for the vascular outcomes, but associations with clustered CVD although of a similar direction to the full analysis presented above, were returned to the null.

## Discussion

To our knowledge, this is the first study to use CoDA models to examine associations between PA and ST and vascular outcomes in a large population sample of children with adjustment for important confounders. Our novel findings are that neither ST, LPA or MVPA were significantly associated with endothelial function, arterial elasticity, or arterial stiffness in the whole group analysis, or with the data stratified by sex. However, time spent in ST was unfavourably associated with the clustered CVD risk at the group level, but this association was only observed in girls in the sex stratified analyses. Additionally, MVPA was favourably associated with clustered CVD risk in the group model, and when stratified by sex. Collectively, these data highlight that in children the associations with activity behaviours are not consistent between measures of arterial structure and function and traditional CVD risk factors, and that some associations may be sex dependent.

Although the proportion of time spent in MVPA was inversely associated with endothelial function and positively associated with arterial stiffness at a group level in the unadjusted models (see Supplementary Table 5), the associations were attenuated to null after the inclusion of key covariates. Our observation of null associations between all activity behaviours (LPA, MVPA and ST) and the vascular outcomes is consistent with previous paediatric literature in children^7,10–12^ and adolescents^8,9^. However, this finding contrasts with the cross-sectional study by Hopkins et al.^52^ who found a favourable association between vigorous PA (but not MVPA) and brachial artery endothelial function in 10 year old children. While this observation suggests performing vigorous intensity PA may be important for promoting endothelial function in children, it should be noted that this observation was only found in a small number of participants (*n*=43) in the lowest tertile for endothelial function with no further adjustment for key covariates, including other activity exposures. Additionally, although Haynes et al.^53^ reported no association between self-reported PA trajectories during childhood and adolescence and upper or lower limb artery function in adulthood, they observed that females exposed to a lower amount of television viewing during childhood and adolescence had superior lower limb vascular function in adulthood. While this observation in females persisted even after adjustment for important CVD risk factors, the model was not adjusted for PA.

Evidence regarding relationships between PA with arterial stiffness or compliance in children is conflicting and likely related to several methodological factors. Akin to the present study, Reed et al.^54^ reported questionnaire determined total PA was not associated with small or large vessel compliance in 99 children aged 9–11 years. By contrast, a cross-sectional study of 160 children aged 6-8 years reported no associations between questionnaire determined total PA and arterial stiffness, but a beneficial association for time spent performing unstructured PA^10^. However, this relationship for unstructured PA was no longer significant once CRF and body fat percentage was considered in the model. A similar observation was reported by Heil and colleagues^55^, where device-determined VPA, but not LPA and MPA, was beneficially associated with arterial stiffness in 80 children aged 8–11 years. However, with further adjustment for important confounders, including BMI, age, sex and accelerometer wear time, the beneficial association for VPA was diminished. While these findings, alongside the data from the current study, underscore the importance of controlling for key confounders, it is important to realise that beneficial associations between total accelerometer determined MVPA and arterial stiffness have been observed in cross-sectional studies, even after adjusting for several covariates^11,12^. While the study by Nettlefold et al.^12^ did not adjust for other activity exposures or CRF, the adjustment for these important covariates was undertaken by Haapala et al.^11^, with their data supporting beneficial associations between MPA or VPA and arterial stiffness (via finger plethysmography) in 6–8 year olds (*n*=136). However, the mutual adjustment for other activity exposures was not undertaken within a compositional framework, meaning the co-dependent nature of the activity exposures were not accounted for^18^.

While there were no significant associations between ST with measures of arterial structure or function, a significant unfavourable association was observed between ST and clustered CVD risk in children. Similar findings to the present study have been observed between ST and CVD risk factors when using a compositional framework in paediatric popultions^19–21^. ST was unfavourably associated with waist circumference in 6-17 year olds, although the association with BMI was inconsistent between studies^19,20^. Interestingly, the same studies observed no significant associations between ST and blood biomarkers for CVD, such as TAG and insulin, indicating the associations for ST are not consistent across all CVD risk factors. However, consistent with the current study, Matriccaini et al.^21^ observed unfavourable associations between ST and a clustered CVD risk score, comprising a measure of adiposity, blood pressure and blood biomarkers, in 1073 12 year old children. Collectively, the studies employing a compositional framework highlight the importance of minimising ST to improve CVD risk in children and adolescents. This contrasts with previous studies where no association between ST and CVD risk factors was found in children and adolescents after adjusting for MVPA^4,56,57^. Finally, it is important to highlight that in the current study the overall group effect for the unfavourable association between ST and CVD risk is likely driven by the significant association with CVD risk in girls, as no association was observed in the boys. This finding suggests the association between ST and CVD risk may be sex dependent, which may be explained by sex differences in either the duration, pattern and/or type of sedentary behaviours. It is difficult to contrast this observation with the literature, as analyses are typically performed at a group level and with adjustment for sex in the regression model^19–21^. However, it has recently been shown that television watching, a specific type of sedentary behaviour contributing to total ST, during childhood and adolescence is predictive of lower limb vascular function measured in adult in females but not males^53^.

Significant favourable associations were found in the present study between the proportion of time in MVPA and clustered CVD risk both at the group level, and when stratified by sex. However, no significant associations were observed for LPA and CVD risk. The importance of MVPA for modifying CVD risk is supported by previous research using a non-compositional approach to account for other activity behaviours such as ST. For example, Ekelund et al.^4^ observed beneficial associations between MVPA and several CVD risk factors in a large sample (*n*=20,871) of children and adolescents aged 4–18 years, where ST was mutually adjusted for in the model as a covariate. Similar results to the current study are also supported by previous studies using a compositional approach. For example, in their fully adjusted models accounting for puberty, sex, season and socioeconomic status, MVPA was favourably associated with a clustered CVD risk in 1,073 12 year olds, while no effect was observed for LPA^21^. Carson et al.^19^ reported favourable associations for MVPA with a range of CVD outcomes, including BMI, waist circumference, blood pressure, TAG and insulin in 6–17 years, which are consistent with the present study. However, they reported unfavourable associations between LPA and markers of body composition (BMI and waist circumference) and systolic blood pressure, but null associations with blood biomarkers, such as TAG and insulin. In a subsequent study on 2,544 6–17 year olds from the USA, Carson et al.^20^ only found an association between LPA and diastolic blood pressure, but no associations for other CVD risk factors, including markers of body composition and blood biomarkers. Therefore, while paediatric studies using a compositional framework support beneficial associations between MVPA and CVD risk factors, the associations between LPA and CVD risk factors are currently equivocal.

A novel finding in the current study is that while no significant associations were observed between activity behaviours and measures of vascular structure and function, significant associations were observed between ST and MVPA with CVD risk factors. This finding may be considered surprising, as previous studies have shown that vascular structure and function in children and adolescents are unfavourably associated with CVD risk factor status^2^. Thus, it may be expected that measures of vascular structure and function and CVD risk factors have similar associations with activity behavours. However, data from the ALSPAC cohort have reported that both fat and lean mass have favourable associations with artrial structure and function in children aged 9-11 years old^58^, suggesting that the adaptation of the vasculature at this early age may reflect physiological remodelling rather than the early stages of disease pathology. The transition from childhood to adolescence and into adulthood is characterised by a tracking of CVD risk factors^59,60^. In addition, CVD risk factor status or the progression of CVD risk factors in childhood and adolescence are predictive of measures of arterial health in adulthood^61–63^, although recent evidence suggests these associations may be bi-directional and that changes in arterial outcomes may proceed changes in CVD risk factors^62,64^. Therefore, it could be hypothesised that prospective changes in activity behaviours from childhood into adolescence may lead to stronger associations with arterial outcomes. Indeed, previous prospective data have shown favourable associations with PA and FMD and aortic intima media thickness in adolescents across a 5 year period^65^, and that PA (especially vigorous PA) in late adolescence (9-15 years old) is favourably associated with vascular outcomes measured in adulthood^66,67^. However, these studies all employed subjective (e.g. questionnaire or interview) based measures of PA, which will be prone to reporting bias and have limited measurement properties when compared to device-derived measures^6^. This underscores the importance of future research to examine prospective associations between device-derived activity behaviours, CVD risk factors and vascular outcomes, especially during adolescence where levels of PA decline and ST increase.

### Methodological considerations

This study has several important strengths. Firstly, the study utilised data from a large cohort study (ALSPAC) which provided a comprehensive suite of vascular outcomes quantifying both arterial structure and function. Nevertheless, it is necessary to note that by restricting the sample to those who had complete vascular data at age 10 and valid accelerometer data at age 11, we had a subsample in the present study that were more likely to be female and engaged in greater amounts of MVPA than the wider ALSPAC sample which may have modified the findings. Importantly, PA and ST were determined using accelerometery, and the analysis of the relationship between activity exposures and the outcomes were undertaken within a compositional framework, which addresses collinearity issues associated with the mutual adjustment for all parts of the activity composition. However, non-waking behaviour was not considered within the compositional framework because data were unavailable. Therefore, not being able to consider the complete 24 h cycle may have masked true associations between activity behaviours and vascular outcomes. Additionally, several important covariates were included in the analytical models to control for the influence of other confounding variables (e.g. puberty, CRF) which may have masked or inflated the observed associations, made possible by the large sample size. The sensitivity analysis performed on the complete cases revealed similar findings, although the associations with the clustered CVD risk factors were returned to the null, likely from the small sample size. Analyses were also presented at a group level and stratified by sex, which provides a significant advancement in reporting of the outcomes compared to previous literature. However, we did not consider the influence of ethnicity as the ALSPAC sample is predominantly White^23^, so future research should investigate whether these findings hold in ethnically diverse samples. It would also be valuable to conduct research in developing countries because the existing data are currently predominantly from developed countries in Europe. Furthermore, it was beyond the scope of the present investigation to consider mediator or moderator variables such as socioeconomic status or diet so future research should examine the potential roles of mediator and moderator variables on the associations between activity behaviours and vascular outcomes.

While the use of accelerometery to quantify PA and ST is a strength of the current work when compared to previous literature, the accelerometer data were summarised over 60 s epochs which is known to underestimate MVPA and overestimate LPA in paediatric populations^68^. As experimental evidence suggests that performing short bouts of activity at both a moderate and vigorous intensity may be important for promoting vascular function^69^, it is possible that the underreporting of MVPA may have biased associations with the vascular outcomes towards the null. Furthermore, the activity behaviour data was not collected at the same time point as the vascular measures or CVD risk factors. This was accounted for in the statistical models but the small time difference between measures may have resulted in some errors in the reported associations if the PA and ST levels of some the children changed between the measurement periods. Although PA has been shown to track in childhood between primary and secondary school (*r*=0.55)^70^, observed associations may be confounded by changes in habitual PA between exposure and outcome measurements not accounted for in these analyses.

A strength of the current study is the measurement of vascular endothelial function using FMD, which has well-described methodology and measurement error within this cohort^32^. Whilst common place and clinically important, FMD was measured at the brachial artery. However, it is possible that the measurement of FMD at the brachial artery may have missed limb specific adaptation to PA and ST. Acute experimental evidence has shown a transient decline in superficial artery FMD in prepubertal girls from 3 hours of prolonged sitting^71^. Adult evidence has also shown the FMD response to prolonged sitting to be limb specific^72^. There is no significant relationship between FMD in the upper and lower limb in adults^73^, therefore, the absence of a significant association between the activity behaviours and brachial FMD in the current study may not mean there is not a significant association between the activity behaviours and FMD in the lower limb. Consistent with the possibility of limb-specific effects are the data of Haynes et al.^53^ who observed trajectories of television viewing in females to be associated with femoral artery FMD but not brachial artery FMD. This limb-specific adaptation to activity behaviours could also apply to arterial stiffness because the present study measured PWV across the carotid-radial sites, rather than using the gold-standard carotid-femoral sites, so the measure did not span the lower limb. Future research should re-examine the associations between the activity behaviours with FMD in the lower limb and carotid-femoral PWV to consider limb specific adaptations to activity.

## Conclusion

Although this study found no significant associations between any of the activity behaviours with the vascular outcomes, the presence of unfavourable associations between ST and CVD risk and favourable associations between MVPA and CVD risk respectively support interventions that promote MVPA and limiting ST in childhood for health benefits. An important sex-specific association between ST and CVD risk was also identified, which will need confirming in future studies. Finally, future studies are required to prospectively examine activity behaviours during the transition from childhood into adolescence, where continued exposure to CVD risk factors and the well document decline in PA and increase in ST, may uncover important associations between PA and ST with vascular outcomes.

## Supplementary Information

Supplementary Information.

## Data Availability

The informed consent obtained from ALSPAC participants does not allow the data to be made freely available through any third-party maintained public repository. However, data used for this submission can be made available on request to the ALSPAC Executive. The ALSPAC data management plan describes in detail the policy regarding data sharing, which is through a system of managed open access. Full instructions for applying for data access can be found here: http://www.bristol.ac.uk/alspac/researchers/access/. The ALSPAC study website contains details of all the data that are available (http://www.bristol.ac.uk/alspac/researchers/our-data/).
