# Supplementary material for: Cross-sectional associations between physical activity and sedentary time with cardiovascular health in children from the ALSPAC study using compositional data analysis
Source: Sci Rep. 2025 Apr 7;15:11878. doi: 10.1038/s41598-025-95407-x (PMC11977000; doi:10.1038/s41598-025-95407-x)
Supplement: Supplementary file 1 — Supplementary Information. [file 41598_2025_95407_MOESM1_ESM.pdf]

Sansum et al. Cross-sectional associations between physical activity and sedentary time with cardiovascular health in children from the ALSPAC study using compositional data analysis – Supplementary file:

Table S1 - Listwise exclusion of missing data

Variable	Valid data available (*n*)	Missing data (%)
Age at 9-year clinic (y)	4089	4.4
Age at 10-year clinic (y)	4277	0
aPHV at 9 (y)	3370	21.2
aPHV at 10 (y)	3449	19.4
Body mass at 9 (kg)	4080	4.6
Body mass at 10 (kg)	4261	0.4
Stature at 9 (cm)	4043	5.5
Stature at 10 (cm)	4247	0.7
BMI at 10 ($kg{\cdot}m^{-2}$)	4240	0.9
BMI-SDS at 10	4240	0.9
Mother's social class	1950	54.4
**Body composition measures at 9 year clinic**		
Total body fat mass (kg)	3903	8.7
Fat mass index ($kg{\cdot}m^{-2}$)	3863	9.7
Total body lean mass (kg)	3903	8.7
Lean mass index ($kg{\cdot}m^{-2}$)	3863	9.7
**Fitness measure at 9 year clinic**		
PWC_{170} (W)	2084	51.3
PWC_{170}·total body mass ($W{\cdot}kg^{-1}$)	2081	51.3
PWC_{170}·total body mass ($W{\cdot}kg^{0.237}$)	2081	51.3
PWC_{170}·total body lean mass ($W{\cdot}kg^{0.585}$)	1991	53.4
**Metabolic profile at 9 year clinic**		
Cholesterol ($mmol{\cdot}L^{-1}$)	2778	35.0
HDL ($mmol{\cdot}L^{-1}$)	2778	35.0
Total cholesterol:HDL ratio	2778	35.0
TAG ($mmol{\cdot}L^{-1}$)	2778	35.0

Insulin (mU·L^{-1})	2760	35.5
Cardiometabolic risk score	2546	40.5
**Family history of hypertension, diabetes, high cholesterol and vascular disease**	2744	35.8
**Vascular measures**		
Systolic blood pressure age 9 (mmHg)	4045	5.4
Diastolic blood pressure age 9 (mmHg)	4046	5.4
MAP age 9	4045	5.4
Systolic blood pressure age 10 (mmHg)	4277	0
Diastolic blood pressure age 10 (mmHg)	4277	0
Baseline vessel diameter (mm) age 10	4277	0
FMD absolute (mm) age 10	4277	0
FMD (%) age 10	4277	0
DC (% per mmHg) age 10	4277	0
PWV (m·s^{-1}) age 10	4277	0
**Accelerometer measures at 11 year clinic**		
Accelerometer wear time (min·day^{-1})	4277	0
ST (min·day^{-1})	4277	0
LPA (min·day^{-1})	4277	0
MVPA (min·day^{-1})	4277	0
Time between visits (y)	4277	0
Time between CMR score and vascular visits (y)	4089	4.4
Time between CMR score and accelerometer visits (y)	4089	4.4

aPHV = age in years from peak height velocity; BMI = body mass index; BMI-SDS = body mass index standard deviation score; PWC_{170} = peak work capacity at 170 beats per minute; HDL = high density lipoprotein; TAG = triglyceride; MAP = mean arterial pressure; FMD = flow mediated dilation; PWV = pulse wave velocity; DC = distensibility coefficient; ST = sedentary time; LPA = light physical activity; MVPA = moderate-vigorous physical activity.

Table S2 - Variables included in the multivariable multiple imputation model

Variable	Missing values imputed?	Imputed (n)	Imputed values	
			Minimum	Maximum
Age at 9-year clinic (y)	Yes	188	8.8	11.6
Age at 10-year clinic (y)	NA			
Sex	NA			
aPHV at 9 (y)	Yes	907	-7.7	0.5
aPHV at 10 (y)	Yes	828	-6.6	1.8
Body mass at 9 (kg)	Yes	197	19.4	71.4
Body mass at 10 (kg)	Yes	16	27.9	63.8
Stature at 9 (cm)	Yes	234	118.1	162.7
Stature at 10 (cm)	Yes	30	124.5	156.3
BMI at 10 ($kg \cdot m^{-2}$)	Yes	37	13.7	29.4
BMI-SDS at 10	Yes	37	-2.0	3.2
Mother's social class	Yes	2327	1	6
**Body composition measures at 9 year clinic**	Yes			
Total body fat mass (kg)	Yes	374	1.43	31.93
Fat mass index ($kg \cdot m^{-2}$)	Yes	414	0.79	15.07
Total body lean mass (kg)	Yes	374	15.64	37.71
Lean mass index ($kg \cdot m^{-2}$)	Yes	414	9.88	16.95
**Fitness measure at 9 year clinic**				
PWC_{170} (W)	Yes	2193	36	88

PWC_{170}·total body mass (W·kg^{-1})	Yes	2196	0.8	3.2
PWC_{170}·total body mass (W·kg$^{0.237}$)	Yes	2196	16.7	37.0
PWC_{170}·total body lean mass (W·kg$^{0.585}$)	Yes	2286	5.9	13.7
**Metabolic profile at 9 year clinic**				
Cholesterol (mmol·L^{-1})	Yes	1499	2.44	7.97
HDL (mmol·L^{-1})	Yes	1499	0.44	2.88
Total cholesterol:HDL ratio	Yes	1499	1.35	10.00
TAG (mmol·L^{-1})	Yes	1499	0.18	4.82
Insulin (mU·L^{-1})	Yes	1517	0.20	2730.00
Cardiometabolic risk score	Yes	1731	-1.74	2.96
**Family history of hypertension, diabetes, high cholesterol and vascular disease**	Yes	1533	1	2
**Vascular measures**				
Systolic blood pressure age 9 (mmHg)	Yes	232	74	145
Diastolic blood pressure age 9 (mmHg)	Yes	231	41	79
MAP age 9	Yes	232	56	98

Systolic blood pressure age 10 (mmHg)	NA			
Diastolic blood pressure age 10 (mmHg)	NA			
Baseline vessel diameter (mm) age 10	NA			
FMD absolute (mm) age 10	NA			
FMD (%) age 10	NA			
DC (% per mmHg) age 10	NA			
PWV (m·s^{-1}) age 10	NA			
**Accelerometer measures at 11 year clinic**				
Accelerometer wear time (min·day^{-1})	NA			
ST (min·day^{-1})	NA			
LPA (min·day^{-1})	NA			
MVPA (min·day^{-1})	NA			
Time between visits (y)	NA			
Time between CMR score and vascular visits (y)	Yes	188	-0.8	2.2
Time between CMR score and accelerometer visits (y)	Yes	188	0.3	3.3

aPHV = age in years from peak height velocity; BMI = body mass index; BMI-SDS = body mass index standard deviation score; PWC_{170} = peak work capacity at 170 beats per minute; HDL = high density lipoprotein; TAG = triglyceride; MAP = mean arterial pressure; FMD = flow mediated dilation; PWV = pulse wave velocity; DC = distensibility coefficient; ST = sedentary time; LPA = light physical activity; MVPA = moderate-vigorous physical activity.

Table S3 - Participant characteristics using imputed and observed data

**Variable**	**Group**		**Boys**		**Girls**	
	**Observed**	**Imputed**	**Observed**	**Imputed**	**Observed**	**Imputed**
Age at 9-year clinic (y)	9.8 ± 0.3	9.8 ± 0.3	9.8 ± 0.3	9.8 ± 0.3	9.8 ± 0.3	9.8 ± 0.3
aPHV at 9 (y)	-2.8 ± 1.3	-2.8 ± 1.3	-3.8 ± 0.9	-3.7 ± 1.0	-2.0 ± 0.9	-2.0 ± 0.9
aPHV at 10 (y)	-2.0 ± 1.3	-2.0 ± 1.3	-3.0 ± 0.9	-2.9 ± 1.0	-1.2 ± 0.9	-1.2 ± 0.9
Body mass at 9 (kg)	34.3 ± 7.1	34.2 ± 7.1	34.0 ± 6.7	33.9 ± 6.7	34.5 ± 7.4	34.4 ± 7.4
Body mass at 10 (kg)	37.6 ± 8.2	37.6 ± 8.2	37.2 ± 7.8	37.2 ± 7.8	38.0 ± 8.5	38.0 ± 8.5
Stature at 9 (cm)	139.3 ± 6.2	139.3 ± 6.2	139.6 ± 6.0	139.6 ± 6.0	139.0 ± 6.4	139.0 ± 6.4
Stature at 10 (cm)	143.8 ± 6.6	143.8 ± 6.6	143.7 ± 6.3	143.7 ± 6.3	143.8 ± 6.8	143.8 ± 6.8
BMI at 10 ($kg \cdot m^{-2}$)	18.1 ± 3.0	18.1 ± 3.0	17.9 ± 2.7	17.9 ± 2.9	18.2 ± 3.1	18.2 ± 3.1
BMI-SDS at 10	0.27 ± 1.14	0.27 ± 1.14	0.34 ± 1.13	0.34 ± 1.13	0.20 ± 1.14	0.20 ± 1.14
**Mother's social class**						

I – Professional (%)	5.5	5.7	6.5	6.6	4.5	4.8
II – Managerial & technical (%)	36.1	35.8	35.4	35.1	36.6	36.4
III – Skilled, non-manual (%)	38.9	38.3	38.1	37.8	39.6	38.8
III – Skilled, manual (%)	1.5	1.9	0.9	1.2	2.2	2.4
IV – Partly unskilled (%)	14.7	14.8	15.4	15.4	14.1	14.3
V – Unskilled (%)	3.3	3.6	3.6	3.8	3.1	3.3
**Body composition measures at 9 year clinic**						
Total body fat mass (kg)	8.32 ± 4.85	8.29 ± 4.86	7.16 ± 4.56	7.19 ± 4.56	9.38 ± 4.86	9.30 ± 4.91
Fat mass index ($kg \cdot m^{-2}$)	4.22 ± 2.30	4.20 ± 2.29	3.61 ± 2.15	3.62 ± 2.14	4.79 ± 2.28	4.73 ± 2.30
Total body lean mass (kg)	24.40 ± 3.17	24.36 ± 3.19	25.39 ± 2.88	25.26 ± 2.95	23.49 ± 3.15	23.53 ± 3.17
Lean mass index ($kg \cdot m^{-2}$)	12.53 ± 0.97	12.54 ± 1.04	12.97 ± 0.84	12.96 ± 0.93	12.12 ± 0.90	12.16 ± 0.99
**Fitness measure at 9 year clinic**						
PWC_{170} (W)	64 ± 9	64 ± 9	67 ± 8	65 ± 9	62 ± 9	62 ± 9
PWC_{170}·total body mass ($W \cdot kg^{-1}$)	1.9 ± 0.4	1.9 ± 0.4	2.1 ± 0.4	2.0 ± 0.4	1.8 ± 0.3	1.9 ± 0.4

PWC_{170}·total body mass (W·kg$^{0.237}$)	27.8 ± 3.8	27.8 ± 3.9	29.3 ± 3.5	28.4 ± 3.8	26.8 ± 3.7	27.2 ± 3.8
PWC_{170}·total body lean mass (W·kg$^{0.585}$)	10.0 ± 1.3	9.9 ± 1.4	10.3 ± 1.2	9.9 ± 1.4	9.8 ± 1.3	9.9 ± 1.4
**Metabolic profile at 9 year clinic**						
Cholesterol (mmol·L^{-1})	4.27 ± 0.63	4.21 ± 0.81	4.21 ± 0.63	4.16 ± 0.80	4.34 ± 0.63	4.26 ± 0.81
HDL (mmol·L^{-1})	1.41 ± 0.31	1.38 ± 0.37	1.44 ± 0.31	1.41 ± 0.38	1.37 ± 0.30	1.36 ± 0.37
Total cholesterol:HDL ratio	3.17 ± 0.79	3.18 ± 0.93	3.03 ± 0.74	3.08 ± 0.90	3.29 ± 0.81	3.27 ± 0.95
TAG (mmol·L^{-1})	1.12 ± 0.52	1.13 ± 0.61	1.10 ± 0.53	1.11 ± 0.61	1.13 ± 0.52	1.14 ± 0.60
*Insulin (mU·L^{-1})	8.03 (10.39)	8.66 (16.87)	8.01 (11.11)	8.69 (17.03)	8.03 (9.87)	8.65 (16.94)
Cardiometabolic risk score	-0.01 ± 0.61	0.06 ± 0.71	0.00 ± 0.60	0.07 ± 0.69	0.02 ± 0.61	0.04 ± 0.71
**Family history of hypertension, diabetes, high cholesterol and vascular disease**						
Yes (%)	30.2	30.3	29.5	29.7	30.8	30.8
No (%)	69.8	69.7	70.5	70.3	69.2	69.2
**Vascular measures**						

Systolic blood pressure age 9 (mmHg)	102 ± 9	102 ± 10	102 ± 9	102 ± 10	102 ± 9	102 ± 10
Diastolic blood pressure age 9 (mmHg)	57 ± 6	57 ± 6	57 ± 6	57 ± 6	58 ± 6	58 ± 6
MAP age 9	72 ± 6	72 ± 6	72 ± 6	72 ± 6	73 ± 6	73 ± 6
Time between CMR score and vascular visits (y)	0.8 ± 0.3	0.8 ± 0.3	0.8 ± 0.3	0.8 ± 0.3	0.8 ± 0.3	0.8 ± 0.3
Time between CMR score and accelerometer visits (y)	1.9 ± 0.3	1.9 ± 0.3	1.9 ± 0.3	1.9 ± 0.3	1.9 ± 0.3	1.9 ± 0.3

Data presented as mean ± SD or * indicates median (interquartile range). aPHV = age in years from peak height velocity; BMI = body mass index; BMI-SDS = body mass index standard deviation score; PWC_{170} = peak work capacity at 170 beats per minute; HDL = high density lipoprotein; TAG = triglyceride; MAP = mean arterial pressure; FMD = flow mediated dilation; PWV = pulse wave velocity; DC = distensibility coefficient; ST = sedentary time; LPA = light physical activity; MVPA = moderate-vigorous physical activity.

Table S4- Associations between activity behaviours with endothelial function, arterial stiffness, arterial elasticity and clustered cardiometabolic risk using compositional data analysis for accelerometer data (observed data only)

	Group			Boys			Girls		
	β_{ilr1} value (95% CI)	*P*-value	*n*	β_{ilr1} value (95% CI)	*P*-value	*n*	β_{ilr1} value (95% CI)	*P*-value	*n*
**Endothelial function**									
ST: LPA & MVPA	-0.337 (-1.677 to 1.004)	0.62	425	0.239 (-1.693 to 2.172)	0.81	169	-0.442 (-2.340 to 1.456)	0.65	256
LPA: MVPA & ST	0.967 (-0.707 to 2.640)	0.26	425	0.182 (-2.419 to 2.782)	0.89	169	1.135 (-1.164 to 3.434)	0.33	256
MVPA: ST & LPA	-0.630 (-1.579 to 0.319)	0.19	425	-0.421 (-2.085 to 1.243)	0.62	169	-0.693 (-1.904 to 0.519)	0.26	256
**Arterial stiffness**									
ST: LPA & MVPA	0.073 (-0.363 to 0.509)	0.74	425	0.139 (-0.531 to 0.809)	0.68	169	0.050 (-0.548 to 0.648)	0.87	256
LPA: MVPA & ST	0.029 (-0.515 to 0.573)	0.92	425	0.115 (-0.799 to 1.018)	0.80	169	0.009 (-0.714 to 0.733)	0.98	256
MVPA: ST & LPA	-0.102 (-0.411 to 0.206)	0.52	425	-0.253 (-0.832 to 0.325)	0.39	169	-0.059 (-0.440 to 0.321)	0.76	256
**Arterial elasticity**									
ST: LPA & MVPA	-0.006 (-0.028 to 0.015)	0.56	425	-0.017 (-0.049 to 0.015)	0.29	169	-0.002 (-0.032 to 0.029)	0.92	256
LPA: MVPA & ST	0.017 (-0.011 to 0.044)	0.23	425	0.038 (-0.005 to 0.081)	0.08	169	0.007 (-0.029 to 0.044)	0.70	256
MVPA: ST & LPA	-0.010 (-0.025 to 0.005)	0.20	425	-0.021 (-0.048 to 0.007)	0.13	169	-0.006 (-0.025 to 0.014)	0.57	256

**Clustered CMR score**
ST: LPA & MVPA
LPA: MVPA & ST
MVPA: ST & LPA

ST = sedentary time; LPA = light physical activity; MVPA = moderate to vigorous physical activity; CMR = cardiometabolic risk
Models were adjusted for age at 10 y clinic (y; or 9 y when CMR was outcome), sex (in whole group models only), age in years from peak height velocity at 10 year clinic (y; or 9 y when CMR was outcome), mother's social class (I to V), baseline vessel diameter (mm; only when FMD was the outcome), the time between vascular and accelerometer measurements (y) or time between CMR score and accelerometer measurements (y), cardiorespiratory fitness scaled to lean body mass ($W \cdot kg^{0.59}$), the lean mass index ($kg \cdot m^{-2}$), CMR score (apart from when CMR score was the outcome), and family history of hypertension, diabetes, high cholesterol, and vascular disease.

Robust variation matrices of activity behaviour components

	**Group**			**Boys**			**Girls**		
	**ST**	**LPA**	**MVPA**	**ST**	**LPA**	**MVPA**	**ST**	**LPA**	**MVPA**
**ST**	0.000	0.032	0.261	0.000	0.039	0.218	0.000	0.030	0.217
**LPA**	0.032	0.000	0.234	0.039	0.000	0.181	0.030	0.000	0.197
**MVPA**	0.261	0.234	0.000	0.218	0.181	0.000	0.217	0.197	0.000

ST = sedentary time; LPA = light physical activity; MVPA = moderate to vigorous physical activity. The robust variation matrix replaces the standard deviation and provides a spread of the compositional parts pairwise (e.g. between LPA and MVPA), where values closer to zero indicate the parts are more dependent upon each other.
